# Evaluating the Effective Frequency of Neuromuscular Electrical Stimulation to Prevent Joint Contractures in a Rat Model

**DOI:** 10.7759/cureus.94268

**Published:** 2025-10-10

**Authors:** Yuta Sato, Takeya Ono, Yuta Hirose, Wataru Tanaka, Rena Kimoto

**Affiliations:** 1 Department of Health and Welfare, Prefectural University of Hiroshima, Mihara, JPN; 2 Department of Rehabilitation, Matsushita Orthopedics Clinic, Chikushino, JPN; 3 Department of Physical Medicine and Rehabilitation, SOALA Inc., Fukuoka, JPN; 4 Department of Rehabilitation, Takarazuka Hospital, Takarazuka, JPN

**Keywords:** joint contracture, joint fixation, muscle, neuromuscular electrical stimulation, non-weight-bearing, tetanic contraction, twitch contraction

## Abstract

Introduction

Non-weight-bearing hind limb joint fixation causes severe joint contracture, and neuromuscular electrical stimulation (NMES) can prevent this. However, the effective frequency of NMES for preventing joint contractures caused by joint fixation with non-weight-bearing hind limbs is unclear. Thus, we aimed to examine the effective frequency of NMES for joint contracture.

Methods

Right ankle joint fixation and hind limb suspension were performed on 60 rats for one week. The rats were divided into four groups: no NMES (NS group), NMES with a stimulation frequency of 1-50 Hz (1 Hz electrical stimulation (ES) group, 10 Hz ES group (artificial twitch muscle contraction), and 50 Hz ES group (artificial tetanic muscle contraction)). We measured the ankle dorsiflexion angle, the extensibility, and the amount of type I and III collagen of the soleus muscle.

Results

The decreases in the ankle dorsiflexion angle and extensibility of the soleus muscle of the 1-50 Hz ES groups on the last day of the experiment were lower than those in the NS group. The decreases in the ankle dorsiflexion angle and extensibility of the soleus muscle of the 1 Hz and 10 Hz ES groups on the last day of the experiment were lower than those of the 50 Hz ES group. There was no significant difference in the amount of type I and III collagen between the groups.

Conclusion

Artificial twitch muscle contractions induced by NMES were more effective than artificial tetanic muscle contractions caused by NMES in preventing joint contracture.

## Introduction

Joint contracture occurs when normally stretchy tissues, such as muscles, tendons, skin, and ligaments, are replaced by non-stretchy tissues due to reduced joint movement [1]. In the previous study, among elderly people living in long-term care facilities (n = 1914), approximately 41.5% had lower-limb joint contractures [2]. Furthermore, in a previous study including participants diagnosed with knee osteoarthritis (analyzed knees, n = 8,667), approximately 32.5% of knees had flexion contractures [3]. Thus, joint contractures are clinically common. When joint contracture occurs in the lower limb joints, it may adversely affect walking [4,5]; therefore, the prevention of joint contracture is important.

In numerous previous animal studies [6-15], fixation of the knee or ankle joint was used to induce joint contractures. Contractures typically develop after one week of fixation [6-14]. The main cause of joint contracture of the knee and ankle joints after one week of joint fixation is the affected muscle tissue [8,9]. In particular, in the case of joint contracture caused by ankle joint fixation for one week, the percentage of muscle contribution to joint contracture is approximately 84.3% [8], and the muscle extensibility is limited [6]. In clinical practice, joint contracture of the lower limbs also occurs in patients who cannot bear weight during joint fixation, such as inpatients using crutches after a fracture or bedridden patients. In cases of non-weight-bearing with joint fixation, joint contracture is more severe than that caused by joint fixation alone [6,7], and muscle extensibility is further reduced [6]. If joint fixation continued, joint contracture severity progressed [9-13]. Progressive joint contracture is difficult to treat [15]; therefore, it is presumed that the symptom of joint contracture that develops for one week is the mildest and is easy to heal. It is thus necessary to consider effective methods for the prevention of joint contractures developed over one week.

In previous studies [11-14], neuromuscular electrical stimulation (NMES), a method for artificially increasing muscle contraction, was effective in suppressing reductions in muscle extensibility and in reducing the occurrence of joint contractures. Furthermore, the NMES also has a preventive effect for joint contracture caused by non-weight-bearing [14]. When performing NMES on the muscles, there are many settings, such as stimulation intensity, frequency, and stimulation duration. In particular, the stimulation frequency changes the type of muscle contraction. If the stimulation frequency is 1-10 Hz, twitch contraction occurs in humans [16]. In contrast, a stimulation frequency >20-30 Hz causes tetanic contraction of muscles in humans [16]. It is assumed that the preventive effect on joint contracture differs depending on the frequency of NMES. However, this hypothesis has not yet been verified. It has also been reported that the treatment of joint contracture delays recovery when muscle loading is too high [17] and that interventions for joint contracture caused by non-weight-bearing should be evaluated more carefully. It is important to clarify the more effective frequency (Hz) for the prevention of joint contracture caused by non-weight-bearing, because it may allow these patients with a lack of joint movement and non-weight-bearing to be treated the safer NMES.

Therefore, this study aimed to clarify the effective frequency of NMES on joint contractures caused by non-weight-bearing hind limb joint fixation in rats.

## Materials and methods

Animals

**Figure 1:**
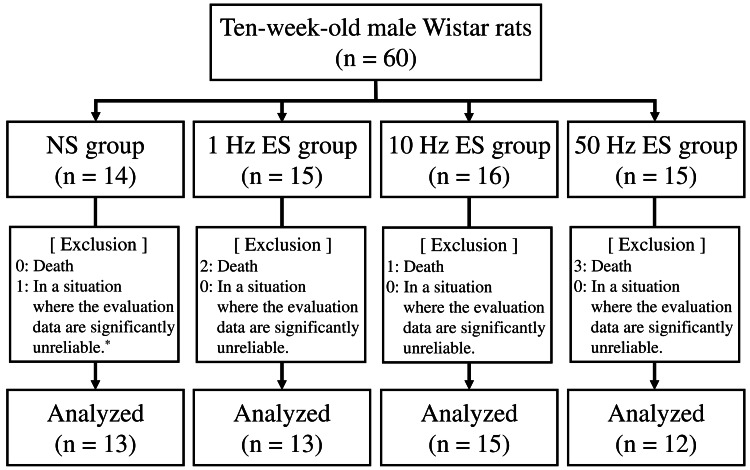
Study flow diagram ^* ^One rat in the NS group is excluded from the analysis because the amount of collagen cannot be measured from 10 fields. NS: no neuromuscular electrical stimulation; ES: electrical stimulation

Ten-week-old male Wistar rats obtained from Charles River Laboratories Japan, Inc. (Kanagawa, Japan) were divided into four groups: (1) non-weight-bearing hind limb joint fixation (NS group; n = 14), (2) non-weight-bearing hind limb joint fixation with an NMES frequency of 1 Hz (1 Hz electrical stimulation (ES) group; n = 15), (3) non-weight-bearing hind limb joint fixation with an NMES frequency of 10 Hz (10 Hz ES group; n = 16), and (4) non-weight-bearing hind limb joint fixation with an NMES frequency of 50 Hz (50 Hz ES group; n = 15) (Figure 1). In common, the sample size of six to nine in each group has been used for animal experiments [6-14]. In this study, the extensibility and histological analysis of the soleus muscle cannot be performed on the same individual animal, because the evaluation of soleus muscle extensibility must extend the muscle over time. Therefore, the sample size in each group in this study was decided to be 14-16 to ensure a sufficient sample size for analysis. All rats were housed in individual cages in a room set at a temperature of 23℃. The room lights were turned on for 12 hours per day (07:00-19:00). The rats were provided ad libitum access to commercially available solid feed and water. All rats were acclimatized to the environment for one week prior to the start of the experiment. The duration of the experiment was one week.

The experimental protocols and breeding of experimental animals were performed in accordance with the Prefectural University of Hiroshima Animal Experiment Regulations, with the approval of the Prefectural University of Hiroshima Animal Experiment Committee (Approval No.: 16A-001-2).

Joint fixation

Under anesthesia of the three-anesthetic mixture of medetomidine (0.375 mg/kg), midazolam (2 mg/kg), and butorphanol (2.5 mg/kg), all rats had their right ankle joints fixed in full plantar flexion for one week using non-elastic tape from the foot to the thigh. The toes of the rats were exposed to confirm the presence or absence of edema, and so that muscle contraction during ES could be confirmed. The non-elastic tape was protected against coming off and damage using a stainless-steel mesh.

Non-weight-bearing

Non-weight-bearing was reproduced in all groups using hind limb suspension [6,7,14]. A Kirschner wire with a diameter of 1.0 mm was inserted into the tail of each rat under anesthesia to form a triangle. A swivel hook was connected to the Kirschner wire triangle. Hind limb suspension was performed by attaching a swivel hook to the ceiling of the cage’s wire mesh.

Neuromuscular electrical stimulation (NMES)

NMES (Pulsecure-PRO KR-7, OG Wellness, Okayama, Japan) was applied to rats once per day in the 1 Hz, 10 Hz, and 50 Hz ES groups under anesthesia for seven days. The rats were released from hind limb suspension and placed lying down on their left side on a plate on which electrodes were affixed. Additional electrodes were percutaneously applied to the right thigh of the rats to stimulate the right soleus muscle through the right sciatic nerve (stimulation intensity, 30 V; stimulation duration, 30 min; and pulse width, 300 µseconds), as described in a previous study [14]. Stimulation frequencies of 1 Hz, 10 Hz, and 50 Hz were applied individually to the 1 Hz, 10 Hz, and 50 Hz ES groups. As the toe flexor muscle group is innervated by a branch from the sciatic nerve in the periphery of the soleus muscle, when ES is properly applied to the sciatic nerve, both the soleus and toe flexion muscle groups contract. This was confirmed by observing the flexion of the right toe.

Measurement of ankle dorsiflexion angle

The range of joint motion was measured on the first and last days of the experiment. Each rat was placed in the left lateral decubitus position under anesthesia, and the right knee joint was fixed in maximum flexion with the trunk to the fixation device using a cable tie, as described in a previous study [6,14]. The hind limbs were supported by an acrylic plate to prevent hip adduction. The head of the right fibula and the lateral malleolus were used as landmarks. A digital tension meter (LTS-1KA; Kyowa Electronic Instruments Co., Ltd., Tokyo, Japan) was used to passively dorsiflex the ankle joint from the metatarsal bone of the rats. The process was video-recorded using a digital camera. As in previous studies [6,7,14], a still image was captured from the video at the moment when a dorsiflexion force of 0.3 N was applied. The ankle dorsiflexion angle, defined as the angle formed by the line connecting the head of the fibula and the lateral malleolus with the line parallel to the bottom of the calcaneus, was measured three times from one still image using image analysis software (Image J, version 1.48v; National Institutes of Health, Bethesda, MD, USA). The mean values were used.

Extensibility of the soleus muscle

**Figure 2:**
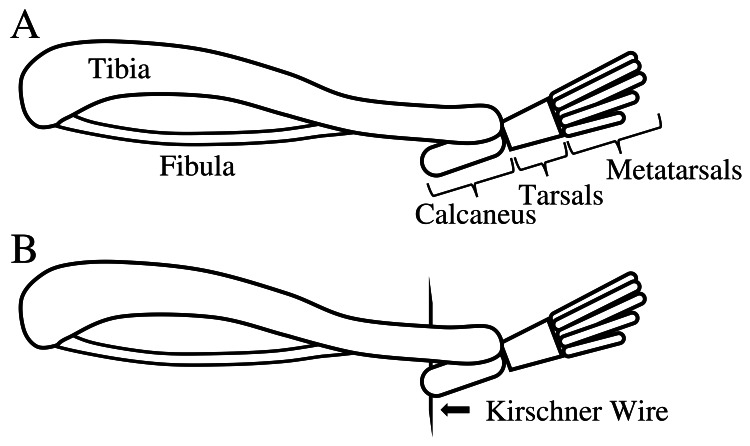
Illustration of the rat ankle joint for the tensile test Illustration of the rat ankle joint: (A) in full plantar flexion; (B) with a Kirschner wire inserted into the tibia and calcaneus to fix the ankle joint in full plantar flexion in preparation for the tensile test. Image credit: The authors.

After measuring the range of joint motion on the last day of the experiment, a tensile test was performed on approximately half of the rats (NS group (n = 6), 1 Hz ES group (n = 8), 10 Hz ES group (n = 8), 50 Hz ES group (n = 6)) in each group to evaluate the extensibility of the right soleus muscle. Kirschner wires (diameter: 0.7 mm) were inserted into the right tibia and calcaneus, and the ankle joint was fixed in full plantar flexion under anesthesia (Figure 2). After sacrifice, the skin from the right hind limb of the rats was excised, the right hind limb was cut from the femur, and a wire was inserted into the tarsal bone. The right hind limb was attached to the tensile tester via femur and tarsal wires. All tissues except the soleus muscle were cut. The soleus muscle tensile test was performed at an extension rate of 10 mm/min using a tensile tester (Autograph, AGS-X 5kN; Shimadzu Corporation, Kyoto, Japan), as described in a previous study [6,14]. The tensile strength was measured at a 10 mm extension, as performed in a previous study [6,14]. Tensile tests were performed within 20 minutes after sacrifice.

Tissue sampling and preparation

After measuring the range of joint motion on the last day of the experiment, the rats in each group that did not undergo tensile testing (NS group (n = 7), 1 Hz ES group (n = 5), 10 Hz ES group (n = 7), 50 Hz ES group (n = 6)) were sacrificed and the right soleus muscle was excised. The excised soleus muscle was frozen in acetone with dry ice and stored in a freezer at -80°C. Using a cryostat to prepare cross-sections, specimens of the soleus muscles were sliced to a thickness of 10 µm and were placed on glass slides for immunofluorescence staining.

Immunofluorescence staining

Tissue cross-sections were fixed with 4% paraformaldehyde. After tissue fixation, the sections were washed three times for five minutes with 0.01 M phosphate buffer in saline (PBS; pH 7.4). The sections were immersed in mouse monoclonal anti-type I collagen primary antibodies (sc-59772; 1:200 dilution; Santa Cruz Biotechnology, Dallas, TX, USA) and a rabbit polyclonal anti-type III collagen (LSL-LB-1393; 1:200 dilution; Life Science Laboratories, Osaka, Japan) diluted in 0.01 M PBS containing 0.4% Triton X-100. The sections with primary antibodies were incubated at 4℃ overnight. After being rinsed in PBS, the sections were immersed in fluorescent-labeled secondary antibodies: Alexa Fluor 488-conjugated anti-mouse IgG (#4408; 1:1000 dilution; Cell Signaling Technology, Danvers, MA, USA) for type I collagen, and Alexa Fluor 555-conjugated anti-rabbit IgG (#4413; 1:1000 dilution; Cell Signaling Technology, Danvers, MA, USA) for type III collagen, diluted in 0.01 M PBS. Secondary antibody incubation was performed at room temperature.

Measurement of fluorescence intensity

Stained sections were photographed at 400× magnification using an all-in-one fluorescence microscope (BZ-X710; Keyence Corporation, Osaka, Japan). Ten fields in the captured images were selected using image analysis software (BZ-X Analyzer; Keyence Corporation, Osaka, Japan). These 10 fields were used to measure the relative area of fluorescence, representing the relative amount of collagen in the muscle tissue. The exposure time and presence of positive areas of type I and type III collagen for each individual section were set using the same image analysis software. Positive areas of type I and type III collagen (µm^2^) were normalized to whole fields (µm^2^), with reference to the methodology described in a previous study [14]. Data were expressed as the positive area of type I or type III collagen divided by the whole field, multiplied by 100 (%).

Statistical analysis

Statistical analysis was performed using the Bell Curve for Excel software (version 3.21; Social Survey Research Information Co., Ltd., Tokyo, Japan). Repeated two-way analysis of variance (ANOVA) was used to compare ankle dorsiflexion angles (dependent variable) with the independent variables: group (NS group, 1 Hz ES group, 10 Hz ES group, and 50 Hz ES group) and time (first day of experiment and last day of experiment). When a group × time interaction was observed, the simple main effect was examined for each independent variable using a post-hoc Tukey’s test. To compare the extensibility and the amounts of type I and type III collagen in the soleus muscle between groups, normality was assessed using the Shapiro-Wilk test. Depending on the distribution, either one-way ANOVA for normally distributed data with post hoc Bonferroni testing, or Kruskal-Wallis testing for non-normally distributed data with post hoc Steel-Dwass tests was performed. The Spearman test was used to analyze the correlation between ankle dorsiflexion angle and the extensibility of the soleus muscle and the correlation between ankle dorsiflexion angle and type I or type III collagen. Statistical significance was set at p-values less than 0.05. The data analyzed by a non-parametric test is shown as median (first to third quartiles), and the data analyzed by a parametric test is shown as mean ± standard deviation in the Results section.

## Results

Ankle dorsiflexion angles

**Figure 3:**
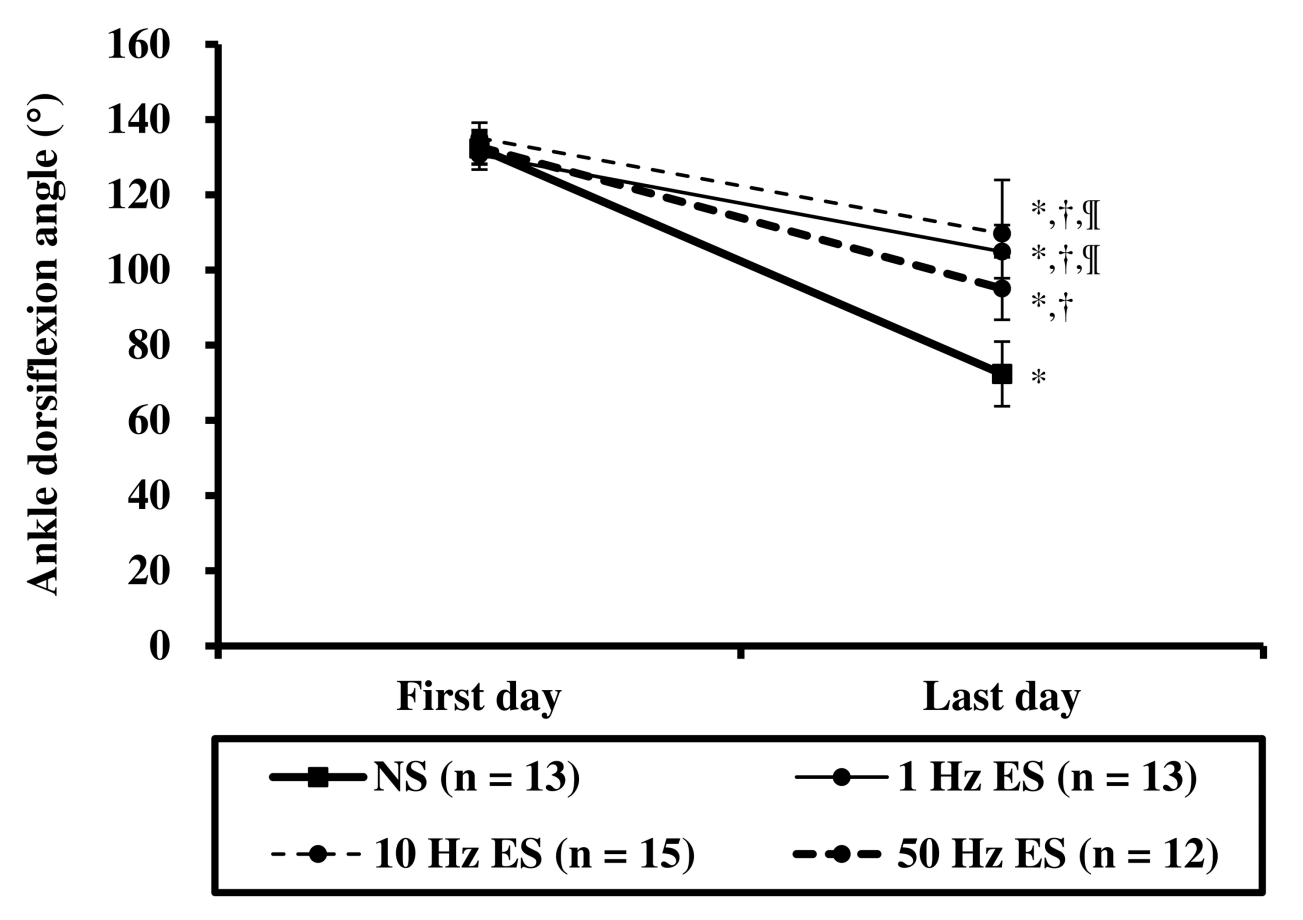
Ankle dorsiflexion angle ^* ^Significant difference compared with the first day (p < 0.05); ^† ^Significant difference compared with the NS group (p < 0.05); ^¶ ^Significant difference compared with the 50 Hz ES group (p < 0.05). NS group: non-weight-bearing hind limb joint fixation; ES: electrical stimulation

The ankle dorsiflexion angles in each group are presented in Table 1 and Figure 3. As a result of repeated two-way ANOVA, the group × time interaction was observed. Therefore, the simple main effect was examined for each independent variable. There was no significant difference in the ankle dorsiflexion angle between all groups on the first day of the experiment; however, there was a significant difference on the last day of the experiment. The ankle dorsiflexion angle in the NS group decreased significantly more than that in the 1 Hz, 10 Hz, and 50 Hz ES groups. The ankle dorsiflexion angle in the 50 Hz ES group decreased significantly more than that in the 1 Hz and 10 Hz ES groups. Significant differences in ankle dorsiflexion angles between measurement times were observed in all groups. The ankle dorsiflexion angle on the last day of the experiment was significantly reduced compared to that on the first day of the experiment.

**Table 1:** Ankle dorsiflexion angle and soleus muscle assessments Sample size A: NS group (n = 13), 1 Hz ES group (n = 13), 10 Hz ES group (n = 15), 50 Hz ES group (n = 12); Sample size B: NS group (n = 6), 1 Hz ES group (n = 8), 10 Hz ES group (n = 8), 50 Hz ES group (n = 6); Sample size C: NS group (n = 7), 1 Hz ES group (n = 5), 10 Hz ES group (n = 7), 50 Hz ES group (n = 6). Data on the ankle dorsiflexion angle and type III collagen are shown as mean ± standard deviation. Data on the tension torque and type I collagen are shown as median (first to third quartiles). NS group: non-weight-bearing hind limb joint fixation; 1 Hz ES group: non-weight-bearing hind limb joint fixation with an NMES frequency of 1 Hz; 10 Hz ES group: non-weight-bearing hind limb joint fixation with an NMES frequency of 10 Hz; 50 Hz ES group: non-weight-bearing hind limb joint fixation with an NMES frequency of 50 Hz. ^a ^Statistical analysis was performed using repeated two-way analysis of variance. ^b ^Statistical analysis was performed using Kruskal-Wallis testing. ^c ^Statistical analysis was performed using one-way analysis of variance. ^* ^Significant difference compared with the first day (p < 0.05); ^† ^Significant difference compared with the NS group (p < 0.05); ^¶ ^Significant difference compared with the 50 Hz ES group (p < 0.05). NMES: no neuromuscular electrical stimulation; ES: electrical stimulation

Measured outcomes		Sample size	Group				X^2^	F-value	P-value
			NS	1 Hz ES	10 Hz ES	50 Hz ES			
Ankle dorsiflexion angle (°)^a^	First day	A	132.3 ± 4.3	130.6 ± 3.9	135.1 ± 4.1	132.7 ± 4.5	‐	31.221 (group × time)	<0.001 (group × time)
	Last day	A	72.4 ± 8.6^*^	104.9 ± 7.1^*,†,¶^	109.7 ± 14.3^*,†,¶^	95.1 ± 8.3^*,†^			
Soleus muscle	Tension torque (N)^b^	B	2.27 (1.79-2.42)	0.15 (0.14-0.22)^†,¶^	0.18 (0.17-0.33)^†,¶^	0.62 (0.50-1.11)^†^	19.837	‐	<0.001
	Type I collagen (%)^b^	C	17.4 (16.3-26.5)	28.5 (23.8-31.5)	15.3 (12.5-17.4)	29.9 (24.8-50.9)	7.154	‐	0.067
	Type III collagen (%)^c^	C	19.3 ± 6.3	15.4 ± 3.4	18.3 ± 5.1	15.7 ± 6.1	‐	0.746	0.537

Extensibility of the soleus muscle

**Figure 4:**
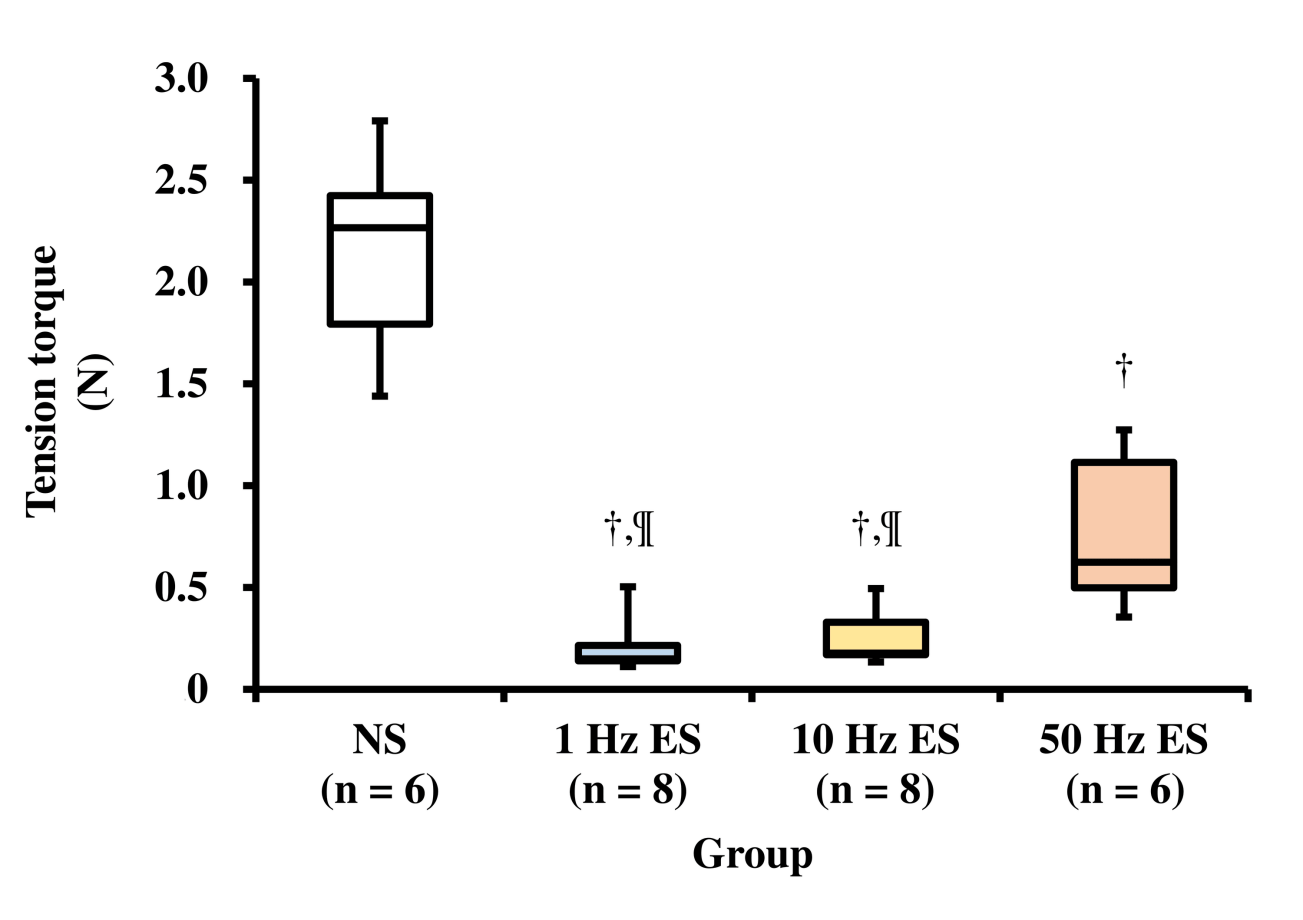
Tension torque of the soleus muscle NS group: non-weight-bearing hind limb joint fixation; 1 Hz ES group: non-weight-bearing hind limb joint fixation with an NMES frequency of 1 Hz; 10 Hz ES group: non-weight-bearing hind limb joint fixation with an NMES frequency of 10 Hz; 50 Hz ES group: non-weight-bearing hind limb joint fixation with an NMES frequency of 50 Hz. ^† ^Significant difference compared with the NS group (p < 0.05); ^¶ ^Significant difference compared with the 50 Hz ES group (p < 0.05). NMES: no neuromuscular electrical stimulation; ES: electrical stimulation

**Figure 5:**
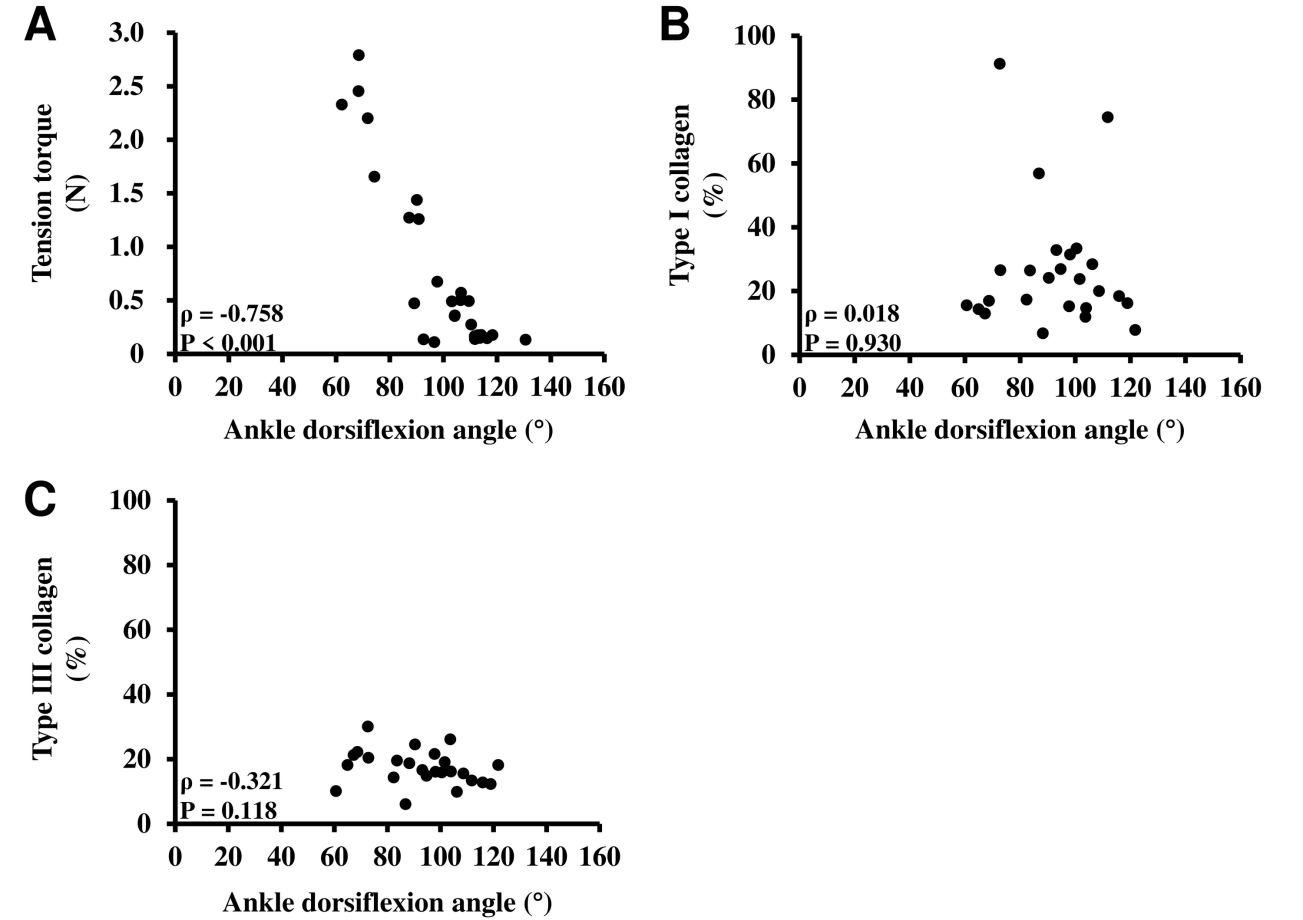
Correlation between ankle dorsiflexion angle and tension torque or type I and III collagen of soleus muscle Statistical analysis was performed using the Spearman test. (A) The correlation between tensile torque and ankle dorsiflexion angle on the last day of the experiment. (B, C) The correlation between type I or III collagen of the soleus muscle and ankle dorsiflexion angle on the last day of the experiment. Correlation coefficients are shown as ρ values. Statistical significance was set at p-values less than 0.05.

The results of the tensile tests on the soleus muscle in each group are presented in Table 1 and Figure 4. The extensibility of the soleus muscle in the NS group was significantly lower than that in the 1 Hz, 10 Hz, and 50 Hz ES groups. The extensibility of the soleus muscle in the 50 Hz ES group was significantly lower than that in the 1 Hz and 10 Hz ES groups. The correlation between the extensibility of the soleus muscle and the ankle dorsiflexion angle is shown in Figure 5. There was a significant negative correlation between the extensibility of the soleus muscle and ankle dorsiflexion angle.

Amounts of type I and type III collagen of the soleus muscle

**Figure 6:**
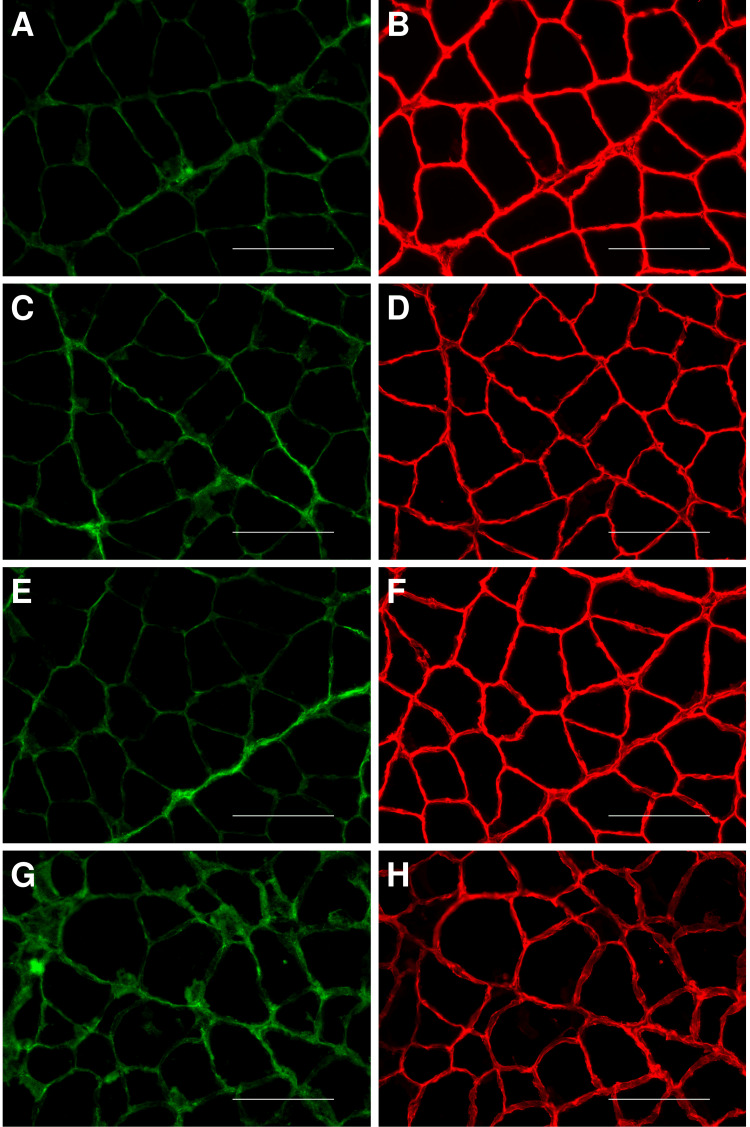
Immunofluorescence staining image of soleus muscle Image magnification is 400. Bar = 100 µm. (A, C, E, G) Images of the soleus muscle stained by immunofluorescence staining for type I collagen. (B, D, F, H) Images of the soleus muscle stained by immunofluorescence staining for type III collagen. (A, B) Images of the soleus muscle in the NS group. (C, D) Images of the soleus muscle in the 1 Hz ES group. (E, F) Images of the soleus muscle in the 10 Hz ES group. (G, H) Images of the soleus muscle in the 50 Hz ES group. NS group: non-weight-bearing hind limb joint fixation; 1 Hz ES group: non-weight-bearing hind limb joint fixation with an NMES frequency of 1 Hz; 10 Hz ES group: non-weight-bearing hind limb joint fixation with an NMES frequency of 10 Hz; 50 Hz ES group: non-weight-bearing hind limb joint fixation with an NMES frequency of 50 Hz. NMES: no neuromuscular electrical stimulation; ES: electrical stimulation

**Figure 7:**
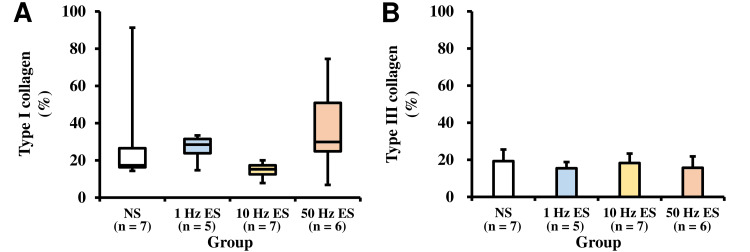
Type I and type III collagen of soleus muscle (A) The type I collagen of the soleus muscle. (B) The type III collagen of the soleus muscle. NS group: non-weight-bearing hind limb joint fixation; 1 Hz ES group: non-weight-bearing hind limb joint fixation with an NMES frequency of 1 Hz; 10 Hz ES group: non-weight-bearing hind limb joint fixation with an NMES frequency of 10 Hz; 50 Hz ES group: non-weight-bearing hind limb joint fixation with an NMES frequency of 50 Hz. NMES: no neuromuscular electrical stimulation; ES: electrical stimulation

The amounts of type I and type III collagen in the soleus muscle of each group are shown in Table 1 and Figures 6-7. There was no significant difference in amounts between the groups. The correlation between type I or type III collagen in the soleus muscle and ankle dorsiflexion angle is shown in Figure 5. There was no significant correlation between type I or type III collagen in the soleus muscle and the ankle dorsiflexion angle.

## Discussion

Joint contracture is caused by joint fixation for one week or more [6-15]; non-weight-bearing lower limbs cause more serious joint contractures than joint fixation alone [6,7]. When joint contracture is caused by non-weight-bearing joint fixation, the levels of type I and type III collagen in the muscle increase [7], and the muscle has decreased extensibility [6]. The main factor of joint contracture with joint fixation in full plantar flexion alone for one week is the muscle; this percentage of muscle contribution to joint contracture is approximately 84.3% [8]. In our study, the ankle dorsiflexion angle in all groups on the last day of the experiment was significantly reduced compared with that on the first day of the experiment, and there was a negative correlation between soleus extensibility and ankle dorsiflexion angle. Therefore, we considered decreased muscle extensibility as the main factor in joint contracture. In contrast, the ankle dorsiflexion angles in the three groups that underwent NMES on the last day of the experiment were significantly higher than those in the NS group. In a previous study, the NMES had a preventive effect for joint contracture caused by non-weight-bearing hind limb joint fixation [14]. Therefore, we concluded that NMES conditions used in this study have a preventive effect on joint contracture caused by non-weight-bearing hind limb joint fixation, as in a previous study [14].

Muscle is composed of muscle fibers and the fascia that surrounds the fibers, such as the perimysium and endomysium [18,19]. The main component of the perimysium and endomysium is collagen [18]. In particular, the amounts of type I and type III collagen in the soleus muscle were increased by non-weight-bearing joint fixation [7]. Therefore, in our study, it was assumed that the amount of type I and type III collagen in the soleus muscle of the NS group was increased by non-weight-bearing hind limb joint fixation. The fascia surrounding the muscle fibers affects muscle extensibility [18]. Therefore, it is assumed that the fascia surrounding the muscle fibers can move with muscle contraction and extension. In the case of ankle joint fixation during full plantar flexion, the soleus muscle is maintained in a short position. Furthermore, in the case of non-weight-bearing hind limbs, soleus muscle contraction is decreased more than that in weight-bearing hind limbs [20,21]. We assumed that the fascia of the soleus muscle could not move much by either joint fixation or non-weight-bearing hind limbs and that the movement of the fascia was facilitated by artificially increasing muscle contractions. Therefore, we considered that the decrease in soleus muscle extensibility in the three groups that underwent NMES was prevented, and ankle dorsiflexion angle in those three groups was higher than that in the NS group. However, there were no significant differences in the amounts of type I and type III collagen between the groups. In a previous study, many normal fascia fibers in the soleus muscle were oriented parallel to its long axis, whereas after joint fixation for four weeks or more, many fibers were oriented parallel to the short axis of the soleus muscle [18]. Therefore, the change in the fascia surrounding the muscle when joint contracture occurs is also a factor in fiber orientation, in addition to the amount of type I and type III collagen. If it is assumed that the change in fascia fiber orientation occurs early due to the severe joint contracture caused by non-weight-bearing hind limb joint fixation for one week, artificial muscle contraction by NMES might prevent muscle extensibility from decreasing with the change in fascia fiber orientation. However, it is unclear whether the fascia fiber orientation was changed, because we did not evaluate this.

Muscle contraction is affected by stimulation frequency; at a stimulation frequency of 1-10 Hz to the muscle, twitch contraction occurs in humans [16]. In contrast, a stimulation frequency >20-30 Hz to muscles causes tetanic contraction in humans [16]. Assuming that muscles in rats are the same as those in humans, it is thought that twitch contraction occurred in the soleus muscle in 1 Hz and 10 Hz ES groups, and tetanic contraction occurred in the soleus muscle in the 50 Hz ES group. In our study, the decrease in the ankle dorsiflexion angle and extensibility of the soleus muscle in the 1 Hz and 10 Hz ES groups was significantly lower than that in the 50 Hz ES group. Therefore, artificial twitch contraction performed by NMES may have a greater preventive effect on joint contracture than tetanic contraction.

Our study found that NMES had a preventive effect on joint contracture caused by non-weight-bearing hind limb joint fixation; in particular, twitch contraction of the soleus muscle had a greater preventive effect than tetanic contraction of the soleus muscle.

This study has some limitations. We did not investigate the muscle fiber orientation. Therefore, it is not known whether the NMES used in this study had any effect on suppressing changes in the muscle fiber orientation. Furthermore, we did not investigate the influence of other ES settings, apart from stimulation frequency. There are many ES settings, such as stimulation intensity, stimulation duration, and pulse width. Therefore, there may be more effective NMES settings with twitch contraction for joint contracture caused by non-weight-bearing joint fixation than the NMES settings used in this study. The results in this study were obtained from the male animals only, and a short experimental period of one week. Therefore, it remains unclear whether gender differences influence or whether NMES affects joint contractures caused by long-term non-weight-bearing hind limb joint fixation.

In future studies, we recommend that muscle fiber orientation, the influence of differences in gender, the effect of NMES for joint contracture caused by long-term non-weight-bearing hind limb joint fixation, and the influence of ES settings other than stimulation frequency should be investigated.

## Conclusions

In this study, we investigated the effective frequency of NMES for preventing joint contractures caused by non-weight-bearing hindlimb joint fixation in rats. The results of this study indicated that artificial twitch muscle contraction using NMES has a greater preventive effect on joint contracture than artificial tetanic muscle contraction using NMES. However, this study did not investigate the muscle fiber orientation and the influence of other ES settings, apart from stimulation frequency, and further studies are required.
